# Targeting NK Cells for HIV-1 Treatment and Reservoir Clearance

**DOI:** 10.3389/fimmu.2022.842746

**Published:** 2022-03-16

**Authors:** Siqin Duan, Shuwen Liu

**Affiliations:** ^1^ Department of Clinical Laboratory, Guangzhou Women and Children Medical Center, Guangzhou Medical University, Guangzhou, China; ^2^ Guangdong Provincial Key Laboratory of New Drug Screening, School of Pharmaceutical Sciences, Southern Medical University, Guangzhou, China; ^3^ State Key Laboratory of Organ Failure Research, Guangdong Provincial Institute of Nephrology, Southern Medical University, Guangzhou, China

**Keywords:** HIV-1, natural killer cells, NK receptors, TLR agonists, antibodies

## Abstract

Combined antiretroviral therapy (cART) can inhibit the replication of human immunodeficiency virus type 1 (HIV-1) and reduce viral loads in the peripheral blood to undetectable levels. However, the presence of latent HIV-1 reservoirs prevents complete HIV-1 eradication. Several drugs and strategies targeting T cells are now in clinical trials, but their effectiveness in reducing viral reservoirs has been mixed. Interestingly, innate immune natural killer (NK) cells, which are promising targets for cancer therapy, also play an important role in HIV-1 infection. NK cells are a unique innate cell population with features of adaptive immunity that can regulate adaptive and innate immune cell populations; therefore, they can be exploited for HIV-1 immunotherapy and reservoir eradication. In this review, we highlight immunotherapy strategies for HIV infection that utilize the beneficial properties of NK cells.

## Introduction

Human immunodeficiency virus type 1 (HIV-1) can be controlled by combined antiretroviral therapy (cART) to the point at which viral loads in the peripheral blood are undetectable. However, the main obstacle to total HIV eradication and a functional cure is the presence of latent HIV-1 reservoirs, which exist in the peripheral blood and lymphoid tissues. To functionally cure HIV-1, several strategies have been proposed, including gene editing technologies based on CCR5, broadly neutralizing antibodies (bNAbs), the “shock and kill” strategy for eradication of shallow reservoirs, and the “block and lock” or “permanent silencing” approach for eradication of deep reservoirs (1). Many latency-reversing agents (LRAs) have been researched as part of the “shock and kill” approach, but only a small number of candidates have been evaluated in clinical trials. Although the majority of LRAs can activate viral transcription, they are unable to induce “killing” effectively. While preventative medications and treatments exist to reduce the disease burden of acquired immunodeficiency syndrome (AIDS), there is no effective cure for HIV; therefore, it is critical to develop useful approaches for HIV immunotherapy.

HIV infects humans through immune cells, including macrophages (2, 3), dendritic cells (DCs) (4), and especially CD4^+^ T cells; this can destroy immune function and result in severe infection, malignant tumors, autoimmune illnesses, and other complications (5). In the past years, the innate immune system has been shown to play a significant role in fighting HIV. The innate immune system is the body’s initial line of defense against foreign pathogens, and it includes a variety of cells and cytokines that fight off pathogens in a nonspecific manner. Among innate immune cells, natural killer (NK) cells play important roles in antiviral and anti-tumor immune responses. Advances in NK cell technology have been spurred by the development of cancer immunotherapy, and these advances have the potential to enhance HIV immunotherapy and to overcome some of the problems with strategies targeting T cells. Numerous data and literature demonstrate the efficacy of NK immunotherapy against tumors (6–9) and liver inflammation (10) have been discussed elsewhere (6–9). In the present paper, we will review recent advances in NK cell technology and the potential of NK cells for use in HIV immunotherapy.

## Overview of NK Cells

Natural killer cells are components of the innate immune system (11) that are phenotypically comparable to T cells, particularly CD8^+^ T cells (2). Human NK cells are derived from CD34^+^ lymphoid lineage cells (HPCs) (12, 13) and found in the liver, peritoneum, placenta, and other organs; they account for 5-15% of peripheral blood mononuclear cells (PBMCs). Immature NK cells in secondary lymph nodes are stimulated by cytokines such as IL-15 and IL-12 from DCs or other antigen presenting cells (APCs) (14) and are subsequently transferred to the peripheral blood, where they gradually acquire cytotoxicity during differentiation and development. NK cells are divided into three or five subgroups according to their expression of CD56 and CD16: CD3^−^CD56^−^CD16^+^, CD3^−^CD56^dim^CD16^+^, and CD3^−^CD56^bri^CD16^+/−^, and the total population of NK cells included these three subsets (9, 15), with the CD56^dim^ NK cells being the most numerous in peripheral blood and the CD56^bright^ NK cells are largely enriched in the liver (16). CD56^bright^ liver-resident NK cells (lrNKs) can be further defined based on the expression of CD69, CD49a, CCR5 and CXCR6 (17). Moreover, technological advances in mass cytometry revealed peripheral blood NK cell diversity with at least 6000 phenotypic populations in an individual assessed by more than 30 parameters simultaneously, which can be used for immunotherapeutic strategies for infection, reproduction, and transplantation (18).

NK cells are multifunctional natural effector cells that have antiviral and antitumor properties. They participate in killing and lysing target cells by recognizing virus-infected cells or tumor cells through their surface receptors. NK cells have diverse mechanisms of killing, including release of cytotoxic granules and apoptosis mediated by the Fas/FasL pathway or TNF-related apoptosis-inducing ligand (TRAIL) (19, 20). NK cells can also engage in antibody-dependent cell cytotoxicity (ADCC) leading to death of antibody-coated cells (21). Finally, NK cells can secrete cytokines and chemokines that contribute to their antiviral effects, including IFN-γ, GM-CSF, TNF-α, CCL3, CCL4, CCL5, XCL1, and XCL2 (14, 17, 18). In addition to their natural killing function, NK cells have significant immunomodulatory roles; for example, they regulate adaptive immune cells such as T and B cells and innate immune cells such as DCs and macrophages to impact the outcome of infections (22, 23). Multiple features of NK cells indicate that they play dynamic roles in immune-mediated protection and homeostasis (24).

## NK Cell Roles During HIV Infection

NK cells play significant roles in HIV-1 infection. In the early stages of viral infection, NK cells act more quickly than adaptive immune cells, and cytotoxic CD56^low^ NK cells expand faster than CD8^+^ T cells (25). HIV infection changes the distribution and functions of NK cell subpopulations (26–28) even after they are partially restored by antiretroviral therapy (ART) (29). Another study found that CD56^low^CD16^+^ NK cells decreased significantly during HIV-1 infection, whereas CD56^high^CD16^+^ NK cells populations were unchanged (26, 30), and this impacted the rapid and early progression of AIDS (31). In addition, The CD56^–^CD16^+^ NK cell subpopulation reduces spontaneous NK cytotoxicity (26). Furthermore, NK cells in HIV-1-exposed seronegative intravenous drug users (HESN-IDU) produce higher levels of IFN-γ and TNF-α (32, 33) compared to NK cells in healthy controls. Another study demonstrated that NK cells can dramatically inhibit viral entry into CD4^+^ T cells and prevent the spread of HIV-1 by producing β-chemokines following stimulation with IL-2 and IL-15 (34). NK cells are also associated with the development of bNAbs during HIV-1 infection (35). Immunogenetics, viral development, and immune escape studies showed that enhanced NK cell activity supported by cytotoxic cytokines and chemokines production are involved in delaying AIDS progression and controlling of HIV infection (36).

“Shock and kill” therapy strategies attempt to reactivate the latent HIV-1 in lymphocytes and tissues by LRAs, and this is followed by killing of the infected cells by the immune system and ART (37). Some classic LRAs have been reported to influence the function and fate of NK cells. For example, the protein kinase C (PKC) and NF-κB activator prostratin induced non-specific NK cell activation and a strong antiviral response, whereas panobinostat decreased NK cell viability, antiviral activity, and cytotoxicity; these factors should be carefully considered when evaluating strategies to eliminate HIV-1 infection (38). In a clinical trial of people living with HIV (PLWH), following analytical treatment interruption (ATI), those treated with panobinostat had decreased proviral HIV-1 DNA levels and increased viral rebound times, and this was correlated with higher frequencies of immunomodulatory CD56^+^ NK cells, CD56^low^ NK cells, and plasmacytoid dendritic cells (39). The impact of panobinostat on NK cell populations may be due to LRA-mediated alterations in germline-encoded NK receptor ligand expression on HIV-infected CD4^+^ T cells. A study of HIV-1 and Hepatitis C Virus (HCV) coinfected individuals receiving cART showed that pegylated interferon-α (PEG-IFNα) induced NK cell activation, and levels of cell-associated proviral DNA were negatively correlated with NK cell quantities (40). Peg-IFN-α2a-mediated HIV-1 suppression was also correlated with NK cell cytotoxicity and innate immune activity (41). These findings indicate that the reduction in HIV-1 reservoirs following treatment with PEG-IFNα occurs mainly due to the antiviral properties of NK cells. It is also worth noting that targeting NK cell subsets to restore any residual dysfunction could enhance their antiviral properties and reduce related comorbidities.

## NK Cell Activation

### NK Cell Receptors

**Figure 1:**
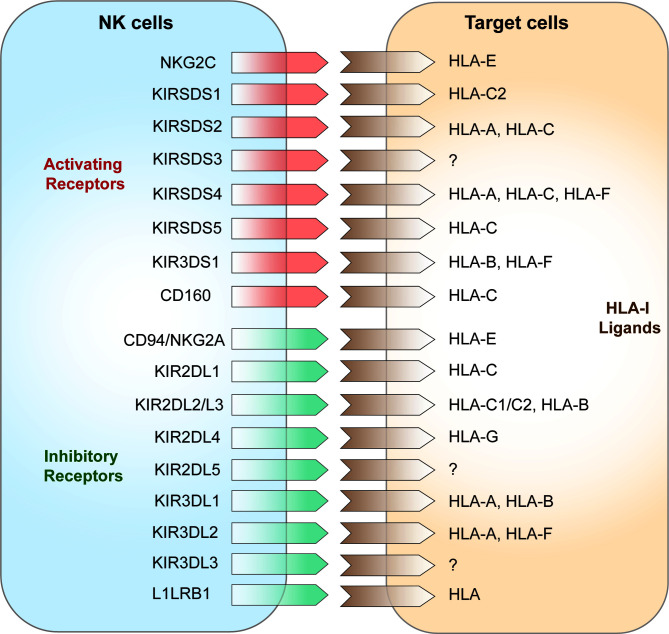
NK cell receptors and HLA class I ligands on target cells.

NK cell surface receptors are divided into two categories according to their structural features: immunoglobulin superfamily receptors (Ig-SF) and C-type lectin superfamily receptors (C1-SF). Ig-SF receptors include i) killer inhibitory receptors (KIRs) and natural cytotoxicity receptors (NCRs), which recognize human leukocyte antigen (HLA)-A, -B, and -C, KIRs are important receptors that regulate the functions of NK cells and are mainly expressed by killer cell subsets. KIRs are also major components of HLA-I binding, which is associated with disease progression (42); and ii) C-type lectin superfamily (C-agglutinin) receptors, chiefly CD94, NKG2, and NKR-P1, which recognize HLA-E (43). According to their functions, NK cell surface receptors can be divided into activating receptors and inhibitory receptors (44). The activating receptors expressed on NK cells are mainly NKG2D, NKG2C, and the NCRs (45, 46); the NCRs include NKP30, NKP44, NKP46 (47). Inhibitory NK cell receptors (iNKRs) include the inhibitory KIRs (iKIRs), LIR1/ILR2, and CD94/NKG2A or CD94/NKG2C heterodimers (**Figure 1**) (8, 48). These iNKRs deliver inhibitory signals to NK cells by recognizing the classical class I molecular antigenic determinants of human leukocyte antigens in “own” target cells, hence preventing attack of “self” cells (49, 50). In humans, KIR3DL1 and KIR2DL1 are two of the prominent NK cell receptors (51). Activation signals *via* NK receptors such as NKp30, NKp44, NKp46, NKG2D, and NKp80 mediate the activation of NK cells consistent with the loss of inhibitory signals (52). Recently a genome-wide CRISPR/Cas9 knock-out strategy identied some unconventional ligands of NK-cell receptors and uncovered a new binding of various KIRs to heparan sulfate proteoglycans that may make an effect on NK cell receptor signaling and target-cell recognition (53). Under healthy conditions, NK cells are not activated when self MHC class I molecules on the target cell surface bind to NK cell inhibitory receptors, which is known as NK cell education [100]. This allows NK cells to distinguish between self and non-self cells. Instead, NK cells are activated and may stimulate activator receptors to release signals and instigate NK cell activities (54) when self cells are transformed or infected with pathogens, and this is followed by subsequent target cell lysis (6).

### Blocking Inhibitory NK Cell Receptors During HIV-1 Infection

HIV-1 infection has been shown to alter NK cell surface receptors (26, 55, 56). The activation status of NK cells is determined by the dynamic balance of activating and inhibitory signals generated by the interactions between NK cell surface receptors and their ligands (57). It has been shown that infection with HIV-1 significantly increases the expression of KIRs on both T and NK cells (58, 59), and the activating receptors NKG2C and CD226/PTA1 on peripheral blood NK cells are abnormally expressed in PLWH (55, 60). A functionally impaired NK cell’s cytotoxicity is related to the decreased expression of NCRs (56). NK cells with a specific transcriptional signature and function reportedly were protective *in vivo* for controlling HIV-1 in CD4^+^ T cells, and increased expression of activating receptor NKp46/NKp30 helped to contain HIV-1 reservoir size (61). The dynamic balance of NK cell activation can also be regulated by interactions with other cells such as neutrophils, macrophages, and dendritic cells, as well as other NK subsets, these cellular interactions also regulate cytokine production, initial viral loads, and CD4^+^ T cell-mediated immune responses during viral infections (62–65). This implies that monitoring, selecting, expanding, and adoptively transferring this NK cell population may be an effective strategy for HIV-1 eradication (61).

However, the quantity and functionality of NK cells in HIV-infected individuals is a matter of ongoing debate. As HIV-1 progresses, continuous stimulation of NK cell receptors may lead to continuous activation, causing chronic inflammation and damage to organs and tissues (15, 66). The expression of HLA class I ligands specific to NK cell receptors changes during HIV infection, for example, higher HLA-A levels render HIV less controllable (67). The downregulation of HLA-C expression on HIV infected cells results in impaired recognition by HLA-C-restricted CTL and a subsequent increase in viral replication *in vitro* (68). NKG2A and inhibitory KIR blockade may also target CD8^+^ T cells and induce a complementary treatment effect. NK cell activity can be inhibited by NKG2A recognizing and binding the ligand HLA-E on target cells (69). Thus, therapeutic blockade of NKG2A: HLA-E interactions may be an effective strategy for HIV eradication.

Motavizumab, an NKG2A blocking antibody, has been studied in various trials to enhance NK and CD8^+^ T cell functionality for cancer immunotherapy (70, 71). Interestingly, it has been shown that blocking the inhibitory receptor NKG2A in mice and patients with chronic hepatitis B virus (HBV) enhances NK cell cytotoxicity and viral clearance (72). Another study demonstrated that an HIV-1 capsid presented by HLA-E can interact with HLA-E specific NK cell inhibitory receptors (iNKRs) NKG2A/CD94, and consequently, NK cells expressing NKG2A can kill the HIV-infected cells (69). However, potential therapeutic approaches that block NK cell inhibitory receptors raise concerns about redundant negative NK cell immunomodulation, unnecessary self-reactivity, and paracellular depletion of activated uninfected T cells (73); these concerns bring challenges for HIV treatment by blocking NK cell inhibitory receptors. Recently research found that HLA-C∗03: 04-presented peptides derived from noninfected CD4^+^ T cells can mediate stronger binding of inhibitory KIR2DL3 than that derived from HIV-1-infected cells, which means that HLA-I-presented peptides alteration induced by HIV-1 infection can reduce engagement of iKIRs and provide a potencial method to activate NK cells by virus-infected cells (74). In summury, blocking iNKRs can enhance NK cell activation and could be of particular benefit for elimination of HIV.

## Enhancing NK Cell Function in HIV-1 Infection

### Antibody-Mediated NK Cell Responses

Freshly isolated PBMCs from HIV-infected patients can kill target cells by ADCC without additional antibodies because NK cells from HIV patients are binding with antibodies against gp120/gp140 and can immediately kill target cells expressing gp120 (75, 76). The ADCC response is an important immune mechanism for host resistance to pathogenic microorganisms and the clearance of diseased cells, and it plays an important role in fighting HIV infection and is thought to be especially important in the small number of long-term slow-progressors (LTSP) (77). In addition to antibodies, NK cells are one of the primary mediators of ADCC because they express CD16, also known as FcγRIII. The NK-mediated ADCC response has been used as an indicator of HIV suppression, and FcγRIII binding and HIV-specific-ADCC activity can be improved by the administration of monoclonal antibodies that are regulated by single nucleotide polymorphisms (SNPs) (78).

NK cells are the main effector cells to perform ADCC, and the overall effectiveness of ADCC in both healthy and HIV-infected individuals relies on NK cells (29). Non-neutralizing functions mediated by interactions with Fc receptors, particularly those expressed by NK cells, may also be required for the eradication of virus-infected cells (79). With the advances of the monoclonal antibodis technique for anti-HIV-1, bNAbs offer stronger antiviral potential and in clearing virus-infected cells with Fc-mediated clearance. In fact, Fc receptor expression levels in humanized mice and rhesus macaques appear to directly impact the ability of bNAbs to inhibit viral replication (80, 81). After passive vaccination, bNAbs have the potential to induce NK-mediated ADCC (82) and hold promise for advancing HIV treatments. Some clinical trials in PLWH have already shown the safety, tolerability, and therapeutic efficacy of bNAb 3BNC117 targeting the CD4 binding site. It has been concluded that 3BNC117-mediated immunotherapy can enhance host humoral immunity to HIV-1 infection (83). In another phase I clinical trial, a virus-like nanoparticles that present clusters of membrane-associated CD4 (CD4-VLPs) bNAbs was highly effective for reducing HIV-1 viremia (84). A separate group found that 3BNC117 has dramatic neutralizing effects *in vitro* and can inhibit several HIV/SHIV strains, and 3BNC117 induces humoral and cellular immune responses *in vivo* to control HIV-1 reservoirs (85). Nevertheless, it will be a challenge to treat 3BNC117-resistant HIV-1 strains using this approach, and the *in vivo* effects of bNAbs on human NK cells remain unknown. Several groups have sought to use NK cells in conjunction with bNAbs to improve targeting of virally infected cells. For example, two bNAbs (3BNC117 and 10-1074) combined with pegylated interferon α2b (peg-IFN-α2b) were used to evaluate ADCC and NK cell activation in PLWH (NCT03588715). Strategies targeting cellular immunity that combine 3BNC117 with other pharmaceuticals (e.g. bNABs, antiretroviral medications, viral inducers) are predicted to benefit the prevention and treatment of AIDS.

### BiKEs and TriKEs

The latent HIV-1 infected cells evade identification and killing by NK cells *via* various immune escape mechanisms. An immunomodulatory strategy has been proposed that brings killer cell engaging molecules into contact with HIV-1 infected cells to boost antigen specificity. These molecules are bispecific and trispecific killer cell engagers (BiKEs and TriKEs) (86), created by connecting an antibody single chain against NK cell CD16 to one or two fragments of a specific antibody against an antigen expressed on the target cell surface. This strategy alleviates many of the issues with NK cell therapy, including lack of specificity in NK cell targeting, limited *in vivo* NK cell activation, viability and proliferation, which can be overcome by higher quality TriKEs and IL-15 supplements. CD4-based BiKEs were demonstrated to activate degranulated NK cells and CD16A-expressing T cells, induce cytokine production, and kill cells expressing HIV-1 envelope glycoproteins (Envs). Due to their small molecular size, high affinity in humans, significant efficacy, and widespread binding to HIV-1 strains, CD4-based BiKEs have enormous potential for curing HIV-1 (87). Clinical trials have examined the combined effects of drugs and antibodies, and it is unclear whether these antibodies act through neutralization effects. Notably, monoclonal antibody-based therapy may facilitate the induction of antibodies directed against such humanized monoclonal antibodies (88), and thus the potential of such antibody-induced hypersensitivity reactions needs to be considered.

## Immune Checkpoint Blockade

Immune checkpoint inhibition can restore the function of exhausted T cells during chronic infection and in the tumor microenvironment; this approach is widely used to treat cancer and could be an appealing adjuvant therapeutic approach for HIV. In addition to iNKRs, NK cells express immune checkpoint receptors previously found on T cells, such as programmed death receptor 1 (PD-1), lymphocyte activation gene-3 (LAG3)/CD223, cytotoxic T lymphocyte associateprotein-4 (CTLA-4), T cell immunoglobulin-3 (TIM-3), T cell immune receptor with Ig and ITIM domains (TIGIT) and the recently identified CD276 (B7-H3) (89). Checkpoint inhibitors may strengthen NK cell activation and cytolytic effectiveness (90). Initial studies of immune checkpoint inhibitors in HIV mainly focused on CD4^+^ T cells. Although checkpoint inhibition has been shown to significantly improve NK cell activity in cancer patients, the benefits of checkpoint inhibition in PLWH on ART have yet to be determined. NK cells, like T cells, can develop an exhausted phenotype during HIV or simian immunodeficiency virus (SIV) infections (91). NK cell exhaustion can cause various problems including poor proliferative capacity, decreased expression of activating receptors (56, 92, 93), and increased expression of inhibitory receptors (26). Furthermore, exhausted NK cells are unable to degranulate, produce cytokines, and promote ADCC (93). PD-1, an key marker of T cell exhaustion during HIV-1 infection, was upregulated on NK cells from HIV-1 positive individuals following ART. Increased PD-1 expression was associated with limited NK cell proliferation, which may impact NK cell maintenance during HIV-1 infection (94). HIV infection and treatment may alter invariant natural killer T cell (iNKT) induced IFN-γ levels. iNKT dysfunction is uniquely associated with the expression of LAG-3, an immune checkpoint marker similar to PD-1, and persistent LAG-3 expression may result in deficient immune reconstitution in PLWH during ART (95).

Multiple activating and inhibitory interactions between T cells and APCs can regulate the immune response. Immune checkpoints promote suppressive interactions between immune cells to maintain homeostasis. Immune checkpoint inhibition has been consistently demonstrated to enhance not only T cell function, but also NK cell function during HIV infection. One group demonstrated that in both untreated and ART-suppressed PLWH, combined PD-1 and IL-10 blockade could enhance NK cell cytokine secretion, degranulation, and killing capacity as well as restore CD4^+^ T cell function. This implies that using immune checkpoint inhibition to improve the cooperation between T and NK cells is a potential therapeutic approach for HIV (96). Therefore, immunotherapeutic interventions targeting immune checkpoint could augment NK cell activity and function against HIV and PLWH with cancers.

## TLR Agonist-Induced NK Cells in HIV-1 Infection

Toll-like receptors (TLRs) are pathogen recognition receptors (PRRs) that detect and modulate signaling responses based on molecular motifs that are conserved across pathogenic microorganisms. TLRs are mainly found on APCs and epithelial and endothelial cells (97), and they are localizated in cell surface (TLR1, 2, 4, 5, 6, 10, 11) and endosomal (TLR3, 7, 8, 9, 12, 13) (98). TLR2 heterodimerizes with TLR1 or TLR6 to function. TLR agonists have been studied as LRAs with the potential to reverse latent HIV-1 and enhance antiviral responses through immunomodulation. TLR agonists enhance immune activation, adaptive responses, and innate antiviral responses *in vitro* and *in vivo* (99). Because of unique immunomodulatory properties of TLR agonists have, further research into the approach to combine latency reversal and viral clearance strategies is undergoing *in vivo* (99). Different TLRs are expressed in NK cells, according to reports, TLR1-TLR9 mRNA was expressed in human NK cells, with TLR1 expression being the highest, followed by moderate levels of TLR2, TLR3, TLR5, and TLR6, and low or undetectable expression of TLR9 levels (100, 101), (reviewed in (102). TLR ligands can activate NK cells directly or indirectly, for example, a new synthetic TLR1/2 ligand named XS15 (103) can stimulate NK cells by monocytes and TLR-7/8 agonist Clo97 can activate NK cells by polymorphonuclear neutrophils (104) indirectly; NK cells also were activated and the cytotoxicity of them were enhanced by autologous DC cultured with poly (I:C) (105). In addition, TLR ligands such as TLR1/2 (Pam3CSK4 or SMU-Z1) or TLR2/6 (MALP-2) and TLR3 (poly I:C) can directly activate human NK cells (106–109). Although poly I:C treatment had no significant effect on CD4^+^ T cells and plasma viral control, the data suggested that poly I:C can induce innate immune responses in PLWH, indicating that it could be a promising adjuvant for HIV therapeutic vaccines (NCT02071095) (110). In SIV-infected rhesus monkeys, TLR7 agonist GS-9620 combined with bNAb PGT121 effectively activated NK cells and CD4^+^ T cells and delayed viral rebound after ART interruption, indicating the potential of NK cell stimulation in conjunction with bNAbs for targeting HIV reservoirs (111). GS-9620 has already been studied in clinical trials NCT03060447 and NCT02858401 in PLWH receiving ART. TLR-7 and/or TLR-8 agonists are being tested for their ability to directly activate NK cells. Although purified NK cells do not express TLR-7 or -8, TLR-7/TLR-8 agonists (R-848) enhance NK-cell cytotoxicity *in vivo* and control NK cell activation indirectly through immune modifiers, and TLR-8 ligands result in indirect NK-cell activation by IL-18 and IL-12p70 and possibly by other cytokines (112). Furthermore, the TLR9 agonist MGN1703 has been shown in clinical studies to activate NK cells in PLWH (113). In summary, TLR agonists have been shown to target cellular and tissue HIV reservoirs in a variety of *in vivo* studies (114). Combined with these results, it is reasonable to expect that TLR agonists will soon become a safe and multipotent clinical drug designed to reduce the HIV reservoir.

## NK Cell Stimulation by Cytokines

Many interleukins such as IL-2, -12, -15, -18 and -21 have been found to boost NK cytotoxicity and proliferation both *in vitro* and *in vivo* (7). In particular, IL-15 is known to promote the proliferation, differentiation, and maturation of NK cells and induces NK cell receptor expression, ADCC, cytotoxicity, and IFN-γ production. After exposure to LRAs, NK cells stimulated with IL-15 were able to kill latent HIV-infected cells (115). Remarkably, the IL-15 superagonist ALT-803/N-803 has been identified as a LRA and can induce HIV elimination in a cooperative manner with CD8^+^ T cells (116). Moreover, in ART-treated macaques, ALT-803 could consistently reactivate SIV in cases of CD8^+^ T cell depletion (117). Furthermore, ALT-803 could be used as an immunomodulator to effectively prevent the formation HIV/SIV: it transiently increased NK cell, CD8^+^ T and memory T cell populations as well as decreased viral loads in SIV-positive rhesus macaques (118). ALT-803 has been evaluated in clinical trials for lymphoma (119), solid and hematological tumors (120, 121), and for synergy with other immunotherapies (NCT01885897). ALT-803 is also undergoing clinical trials to assess the possibility of HIV reservoir elimination (NCT02191098). A phase II, randomized, unblinded, controlled trial will be conducted to investigate the safety, tolerability, and immunomodulatory effect of combining N-803 with ART during acute HIV infection (NCT04505501). The efficacy of N-803 with or without bNAbs is being evaluated for curing HIV in PLWH receiving analytic treatment interruption (ATI) (NCT04340596). There will be more good news to report in the future. ALT-803 has shown significant results in the treatment of bladder cancer and initial success in HIV infection, however, only one drug is not enough to eliminate AIDS and more combinations need to be explored.

## Adoptive NK Transfer Therapy

### Adaptive NK Cells Responses

Classical NK cells are defined as such because they develop antigen receptors irrespective of the recombination activating gene (RAG) (122). *In vivo* studies in mice, nonhuman primates, and humans have indicated that NK cells are capable of quickly responding in an antigen specific manner (123–125). NK cells, like adaptive memory T cells, can acquire memory properties and produce stronger immune responses to previously seen antigens (126–130). Several paradigm-shifting efforts led to the acknowledgement that NK cells have features of adaptive immunity (131, 132). Studies of CD94^+^NKG2C^+^ NK cells in patients infected with cytomegalovirus (CMV) provided the first indications of adaptive-like NK cells (133–135), and memory-like NK cells that respond to CMV have since been identified in mouse models and in humans (136). Observations that NK cells can recall prior immune responses have made NK cell memory a research hotspot. However, numerous questions about NK cell memory remain. It is unclear which NK cell subsets act as “memory” initiators or which ligands induce memory, and it is uncertain if these cells can conduct effector functions immediately upon reactivation by HIV-1 (137). Despite these concerns, it is critical to understand the basic mechanisms underlying NK cell memory in HIV-1 infected patients for improving vaccine-induced cytolytic therapies and eliminating reservoirs (138).

### Haploidentical and Allogeneic NK Transfer

NK cells can be obtained from bone marrow or umbilical-cord blood (UCB), as well as from human embryonic stem cells or induced pluripotent stem cells (iPSC). NK cells can be acquired from patients, which is known as the autologous setting, or from healthy donors, which is known as the allogeneic setting. There are limited circulating NK cells in the peripheral blood, and naturally-derived NK cells have low cytotoxicity; therefore, many strategies have been proposed to expand NK cells to obtain large quantities capable of antitumor and antiviral effects (139, 140), which could allow for adoptive NK cell transfer therapy. For example, PM21 particles from plasma membranes of K562-mb21-41BBL cells, which express membrane bound IL-21 (mb21) and 4-1BB ligand (41BBL), can promote *ex vivo* specific NK-cell expansion from PBMCs in healthy donors and stimulate *in vivo* NK cell expansion in mice, PM21 particles may make NK cell–mediated immunotherapy more widely accessible to HIV individuales (141). Moreover, PM21-NK cells can efficiently kill oncolytic parainfluenza virus 5 (P/V virus) infected cancer cells (142), which support the potential of combining P/V virus with PM21-NK cell adoptive therapy against cancer and HIV. Otherwise, several clinical trials are underway to investigate the effectiveness of NK cell adoptive transfer for treating HIV. Two clinical trials are underway to determine whether haploidentical NK cells combined with the IL-15 agonist ALT-803 or with IL-2 are safe and well-tolerated, and if there is any measurable impact on *in vivo* reservoirs (NCT03899480, NCT03346499).

In addition to haploidentical NK cells, there are several allogenic sources of NK cells for adoptive transfer HIV-1 therapy. UCB, for example, is a robust source of mature, phenotypically and functionally therapeutic effector NK cells without an exact HLA match (143). UCB NK cells produce the same level of IFN-γ and higher levels of perforin and granzyme B than peripheral blood NK cells in response to several stimuli tested (144). Clinical trials NCT01619761 and NCT02280525 are actively evaluating the therapeutic efficacy of umbilical cord blood transplants for treating patients with hematological malignancies. UCB is also a rich source of hematopoietic progenitor cells (HPC)s, which may differentiate into the effector NK cells required for different phenotypes *in vitro*. The clonal NK cell line NK-92 mediates intense cytotoxic responses against a wide range of tumor cells but not against non-malignant healthy cells, and it has been extensively studied in clinical trials for cancer immunotherapy (145–147); therefore, NK-92 cells are expected to become a future therapeutic option for HIV-1. Furthermore, independent of HLA haplotype, stem cell-derived NK cells may be employed as standardized ‘off-the-shelf’ treatments for all donors, allowing for the production of large quantities of homogeneous NK cells that are easier to genetically manipulate than primary NK cells (148). Stem cell-derived NK cells and iPSCs are currently being evaluated clinically in NCT04023071 and NCT04245722 for anti-lymphoma therapy [reviewed in (8)], but they may also have future uses for AIDS therapy.

### CAR-NK Cells

Autologous chimeric antigen receptor (CAR) T cells, an exciting new horizon in anticancer therapy, have shown remarkable clinical efficacy for the management of different haematological malignancies (149–152). However, due to limited clinical trials of CAR-T cells for HIV treatment (117), no significant differences in HIV reservoirs were observed following CAR-T cell therapy. CAR-T cell therapy has been associated with significant side effects, including cytokine release syndrome (CRS) and neurotoxicity, off-target effects, antigen escape, and life-threatening graft-versus-host-disease (GVHD) (153–155). NK cells are an exciting novel candidate platform for CAR-engineering due to their increased safety, modifiability, and superior cytotoxicity. There has been no evidence of the adverse events associated with CAR-T cell therapy when CAR-NK cell therapy has been employed for hematological malignancies (156). Researchers are working to exploit CAR-NK cells derived from the NK-92 cell line, stem cells, or cord blood for the treatment of multiple tumors. In addition, humanized mice treated with both CD4ζ-modified and CD4ζ-unmodified human embryonic stem cell (hESC)-NK or iPSC-NK cells can significantly suppress HIV replication, despite the fact that CD4ζ expression is not associated with increased suppression of HIV (157). Another study reported that CAR-modified HPCs could differentiate into functional NK cells and T cells, which can prevent HIV infection in humanized mice and pave the way for HIV clearance (158). CAR-NK cells can detect and destroy mimetic HIV-infected cell lines, especially primary CD4^+^ T cells expressing gp160 subtypes B and C, making CAR-NK cells a viable approach for HIV eradication (159). Due to their unique characteristics, NK cells are promising candidates for CAR-based cellular immunotherapy in controlling HIV and HIV associated cancers more specifically and efficiently.

## Conclusion

**Figure 2:**
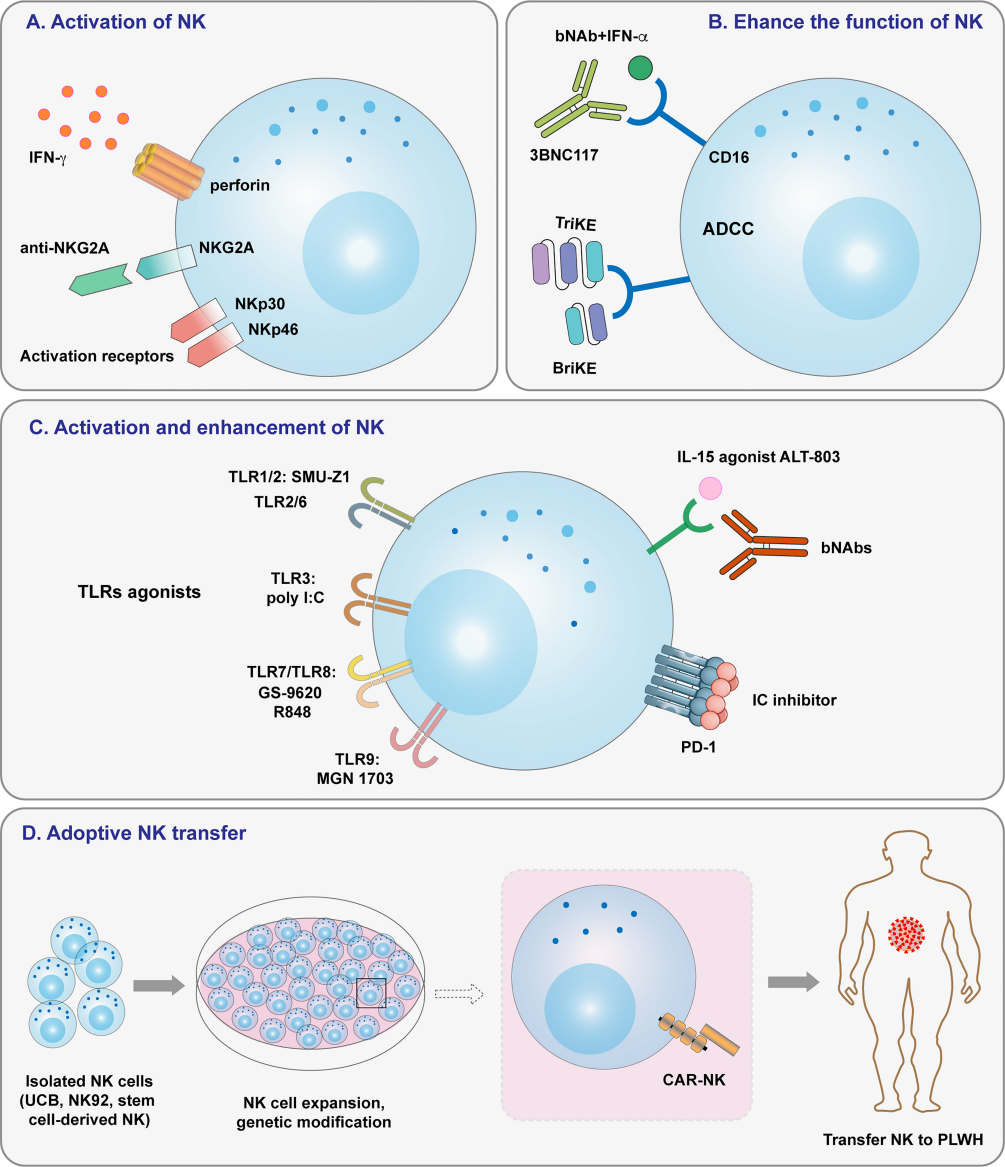
Overview of strategies targeting NK cells for HIV-1 treatment. **(A)** NK cell activation induced by NK cell receptors. **(B)** Enhancing NK cell function by ADCC and specific antibodies. **(C)** Activating and enhancing NK cell activity by TLR agonists, IL-15 agonists, and immune checkpoint inhibitors. **(D)** Adoptive cell transfer, including that of haploidentical NK cells, allogeneic NK cells, and CAR-NK cells.

In addition to T cells, NK cells are an efficient part of the immune response to HIV and act as a bridge between the adaptive and innate immune systems. Several NK cell-based therapeutics are currently used to treat cancer, and it is likely that they will be re-deployed to treat HIV. Some researches has focused on natural killer cell-based HIV immunotherapeutics (53, 74, 88, 160, 161). Herein, we outline several approaches and highlight ongoing studies for treating HIV by enhancement of NK cell activation or cytotoxicity (**Figure 2**). NK cell super-agonists may also be useful for HIV viral suppression and reservoir clearance, and NK cell transfer is another powerful weapon for fighting HIV. CAR-NK cells are advantageous because they do not require HLA-matching and are abundantly available; therefore, they have the potential to be made into off-the-shelf products that can be used immediately and extensively in the clinic. Donor-related variability in NK cell expansion makes the utilization of NK cell lines an efficient and attractive substitute for cell therapy. Strategies that combine NK cell-based immunotherapy with other therapeutic approaches have shown success in numerous of clinical trials. In conclusion, the inherent nature of NK cells will likely make them a crucial tool for future multimodal strategies to clear HIV-1.

## Author Contributions

SL contributed to the conception of the review. SD and SL wrote and revised the manuscript. All authors contributed to the article and approved the submitted version.

## Funding

This work was supported by the National Natural Science Foundation of China (grant 81773787 to SL), the National Science and Technology Major Project (grant 2018ZX10301101 to SL), and the Guangdong Natural Science Foundation Research Team Project (grant 2018030312010 to SL).

## Conflict of Interest

The authors declare that the research was conducted in the absence of any commercial or financial relationships that could be construed as a potential conflict of interest.

## Publisher’s Note

All claims expressed in this article are solely those of the authors and do not necessarily represent those of their affiliated organizations, or those of the publisher, the editors and the reviewers. Any product that may be evaluated in this article, or claim that may be made by its manufacturer, is not guaranteed or endorsed by the publisher.
